# Maternal Diet and Insulin-Like Signaling Control Intergenerational Plasticity of Progeny Size and Starvation Resistance

**DOI:** 10.1371/journal.pgen.1006396

**Published:** 2016-10-26

**Authors:** Jonathan D. Hibshman, Anthony Hung, L. Ryan Baugh

**Affiliations:** 1 Department of Biology, Duke University, Durham, North Carolina, United States of America; 2 University Program in Genetics and Genomics, Duke University, Durham, North Carolina, United States of America; University of California San Francisco, UNITED STATES

## Abstract

Maternal effects of environmental conditions produce intergenerational phenotypic plasticity. Adaptive value of these effects depends on appropriate anticipation of environmental conditions in the next generation, and mismatch between conditions may contribute to disease. However, regulation of intergenerational plasticity is poorly understood. Dietary restriction (DR) delays aging but maternal effects have not been investigated. We demonstrate maternal effects of DR in the roundworm *C*. *elegans*. Worms cultured in DR produce fewer but larger progeny. Nutrient availability is assessed in late larvae and young adults, rather than affecting a set point in young larvae, and maternal age independently affects progeny size. Reduced signaling through the insulin-like receptor *daf-2*/InsR in the maternal soma causes constitutively large progeny, and its effector *daf-16/*FoxO is required for this effect. *nhr-49/*Hnf4, *pha-4/*FoxA, and *skn-1/*Nrf also regulate progeny-size plasticity. Genetic analysis suggests that insulin-like signaling controls progeny size in part through regulation of *nhr-49*/Hnf4, and that *pha-4*/FoxA and *skn-1*/Nrf function in parallel to insulin-like signaling and *nhr-49*/Hnf4. Furthermore, progeny of DR worms are buffered from adverse consequences of early-larval starvation, growing faster and producing more offspring than progeny of worms fed *ad libitum*. These results suggest a fitness advantage when mothers and their progeny experience nutrient stress, compared to an environmental mismatch where only progeny are stressed. This work reveals maternal provisioning as an organismal response to DR, demonstrates potentially adaptive intergenerational phenotypic plasticity, and identifies conserved pathways mediating these effects.

## Author Summary

Information from a mother’s environment can be transmitted to her offspring. In theory, the way mothers provision offspring can be beneficial or pathological depending on whether the environments of the mother and her offspring match. We find that roundworms fed a restricted diet produce fewer but larger offspring. These offspring recover better from starvation, growing faster and having increased fertility. Thus, we find that worms are more likely to thrive after early-life starvation if their mothers have been preconditioned with limited nutrient availability. We describe a genetic network that mediates effects of a mother’s diet on the size and starvation resistance of her offspring. The same genes required to extend the lifespan of worms fed a restricted diet are also required for the differential maternal provisioning we describe. In particular, insulin-like signaling, *pha-4*/FoxA, *skn-1*/Nrf, and *nhr-49*/Hnf4 function in the mother to transmit information about her diet to her offspring. Our work underscores the impact of maternal diet on reproductive health, with consequences for offspring physiology. The conserved genetic network controlling such effects of diet across generations is likely relevant to human diseases related to nutrient sensing and storage.

## Introduction

Developmental physiology can be profoundly influenced by maternal environment. Mismatch between conditions during early development and later in life is thought to contribute to disease [1–3]. For example, children of malnourished mothers have low birth weight and increased risk of diabetes, obesity, and cardiovascular disease [4–7]. Although these effects appear maladaptive, early-life metabolic reprogramming could increase fitness if future conditions are appropriately anticipated. For example, the "thrifty phenotype" is characterized by nutrient rationing and increased fat storage, which in theory is adaptive in poor conditions though posing disease risk in rich conditions [8]. Potentially adaptive maternal effects of environmental conditions have been described for a variety of organisms including the American bellflower, daphnia, beetles, and roundworms [9–13]. It has been reported that *C*. *elegans* fed a restricted diet produce larger embryos and that these progeny are less likely to form dauer larvae, a developmental diapause in the third larval stage caused by high population density, limited nutrient availability, and high temperature [14]. Despite documentation of potentially adaptive maternal effects, molecular mechanisms for intergenerational phenotypic plasticity are generally not understood.

Dietary restriction (DR) increases lifespan in many organisms including *S*. *cerevisiae*, *C*. *elegans*, *Drosophila*, and mammals [15–19]. DR also extends reproductive lifespan in *C*. *elegans*, but it typically reduces total fecundity [19–21]. Insulin-like signaling has pleiotropic effects in *C*. *elegans*, affecting aging, developmental arrest, metabolism, stress resistance, and associative memory [22]. Despite its dramatic effects on lifespan, insulin-like signaling is generally not thought to be required for lifespan extension in DR [23–25]. However, there is substantial variation in conditions used for DR, and insulin-like signaling is involved in certain cases [26,27]. The transcription factors PHA-4/FoxA and SKN-1/Nrf are required for DR to extend lifespan in *C*. *elegans* [28,29]. The *C*. *elegans* nuclear hormone receptor *nhr-49* is an Hnf4α homolog that regulates fat metabolism and the response of the adult hermaphrodite germline to starvation [30–32]. Potential roles of insulin-like signaling, *pha-4*/FoxA, and *skn-1*/Nrf in consequences of DR beyond lifespan have not been examined.

We report here characterization of the maternal effects of DR in *C*. *elegans*. Consistent with previous reports, we show that DR delays reproduction but increases progeny size [14]. We demonstrate maternal effects of reduced insulin-like signaling and show that insulin-like signaling mediates effects of nutrient availability on maternal provisioning, regulating intergenerational phenotypic plasticity. We provide evidence that *nhr-49/*Hnf4, *pha-4/*FoxA, and *skn-1/*Nrf also regulate provisioning in response to nutrient availability. Furthermore, we demonstrate that progeny of DR worms grow faster and are more fertile following extended starvation upon hatching as larvae, as if phenotypic plasticity buffers them from starvation in anticipation of adverse conditions.

## Results

### Dietary restriction reduces adult size and delays reproduction

**Fig 1:**
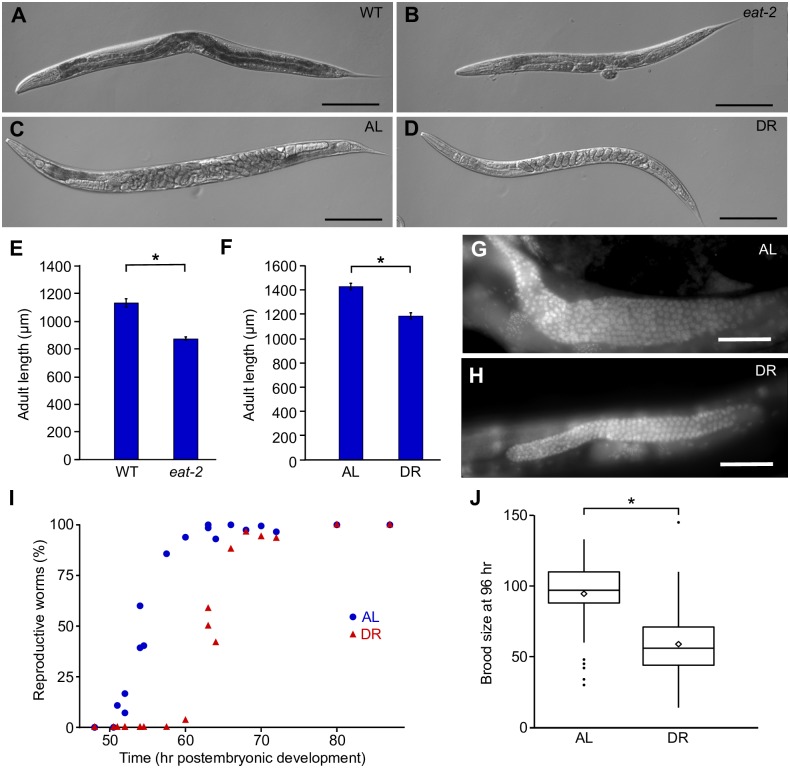
DR reduces adult size and early fecundity. A,B) Representative adult worms after 96 hr in culture for WT and *eat-2(ad465)* are shown. C,D) Representative adult worms cultured in AL or DR for 96 hr (starting from L1 arrest) are shown. Scale bars in A-D are 200 μm. E) Adult length (96 hr after L1 arrest) is plotted for WT and *eat-2(ad465)*. F) Adult length (96 hr after L1 arrest) is plotted for AL and DR. Mean and SEM are plotted in E and F (*p<0.05, paired t-test, n = 3 and 4, respectively). G,H) Representative DAPI-stained worms cultured in AL or DR conditions for 96 hr are shown. Scale bars are 20 μm. I) The average proportion of worms with at least one embryo *in utero* is plotted against time in culture (starting from L1 arrest). Data are from sampling 5 independent biological replicates. J) The number of offspring laid after 96 hr in AL or DR culture is plotted (*p = 0.02, paired t-test, n = 3). Data are pooled and presented as a boxplot reflecting the quartiles. Whiskers extend to the lowest and highest data points within 1.5x the interquartile range. Outliers appear as dots, and diamonds depict the mean value of the pooled data.

We used two different systems to impose DR throughout postembryonic development. Food intake is limited by reduced pharyngeal pumping in *eat-2* mutant worms, providing a genetic model of DR on solid media with *E*. *coli* OP50 as food [33]. Adult *eat-2* mutants appeared smaller and less opaque than adult wild-type (WT) worms, consistent with DR (Fig 1A and 1B). Quantitative image analysis revealed that *eat-2* mutants were 23% shorter than WT at 96 hr of postembryonic development with a 50% reduction in volume (2621 μm^3^ and 5254 μm^3^, respectively) (Fig 1E). These results confirm that the *eat-2* mutant had a significant effect on growth and adult size.

Dilution of bacteria in liquid culture enables careful control of food availability (S1A Fig), providing a second model of DR [34]. Liquid media did not support bacterial growth (S1B Fig), and worms were cultured at low density (1 worm/100 μL), such that that bacterial density remained constant (S1C Fig). Worms fed *ad libitum* (AL) in liquid culture held embryos in the uterus and appeared bloated at 96 hr of postembryonic development (Fig 1C). As gravid adults, worms in DR appeared smaller, and their embryos were more ordered within the uterus (Fig 1D). Similar to *eat-2* mutants, WT worms in DR did not grow to be as large as worms fed AL. Adult worms at 96 hr of postembryonic development in DR were 17% shorter than worms fed AL with a 46% decrease in volume (3766 μm^3^ and 7019 μm^3^, respectively) (Fig 1F). Volume of worms grown in liquid culture is likely affected by the increased number of embryos held *in utero*.

Reproductive development was affected in liquid culture DR. DR reduced the size of the gonad and the number of germ cells it appears to contain (Fig 1G and 1H), as reported [35]. Onset of reproduction was also affected. 50% of worms fed AL had at least one fertilized embryo *in utero* after 54 hr in culture (Fig 1I). DR worms did not reach this point until 63 hr. DR also affected production of early progeny. At 96 hr in liquid culture, AL worms produced an average of 95 progeny, while DR worms produced 58 progeny on average (Fig 1J). Although delayed onset of reproduction presumably limits early fecundity, total brood size is also reduced in some DR models [19,21].

### Dietary restriction increases progeny size

**Fig 2:**
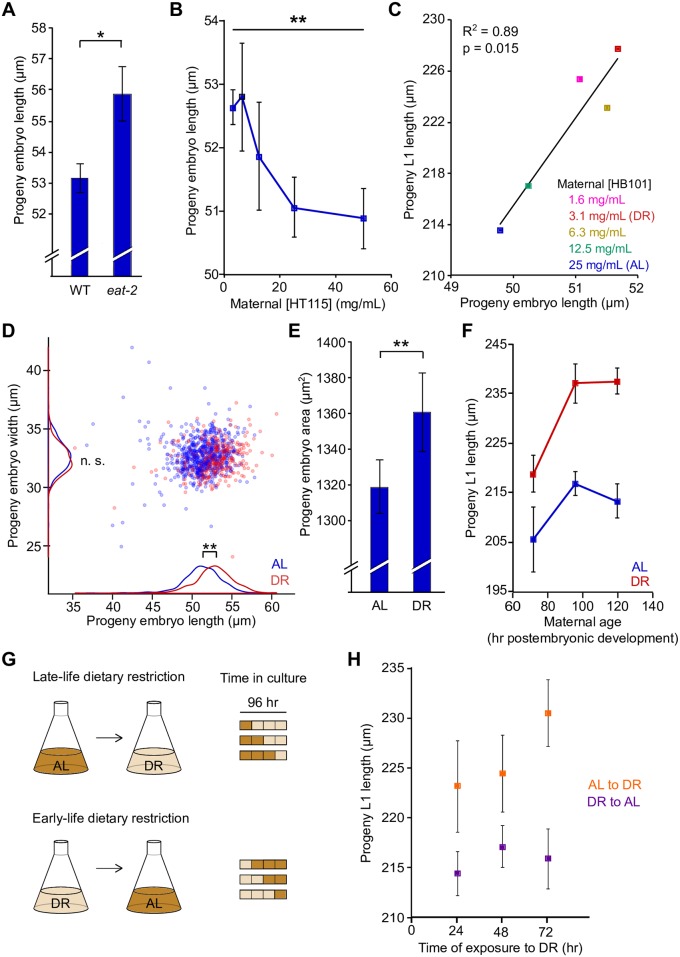
DR increases progeny size. A) Progeny embryo length for WT (N2) and *eat-2(ad465)* (*p = 0.02, paired t-test, n = 4). B) Progeny embryo length as a function of HT115 density (**p = 0.002, ANOVA, n = 5). C) Progeny embryo length is plotted against L1 length across a range of HB101 densities. D) AL and DR progeny embryo length is plotted against width for individual embryos (AL, n = 648; DR, n = 358), with density plotted along each axis. Embryo length is significantly affected (p = 0.0007, paired t-test, n = 7) but width is not (p = 0.28, paired t-test, n = 7). E) Embryo cross-sectional area is plotted for AL (25 mg/ml HB101) and DR (3.1 mg/ml) progeny (**p = 0.006, paired t-test, n = 7). F) Delayed development of DR animals does not explain the progeny size increase. L1 length is plotted for AL and DR progeny as a function of maternal age. Diet and maternal age both affect progeny L1 length (p_diet_ = 0.0001 and p_mat.age_ = 0.006, 2-way ANOVA, n = 3), but there is no interaction between these two factors (p_int_ = 0.41). G) Schematic for experiment in H. H) Progeny L1 length as a function of time of exposure to DR for mothers transferred between conditions. Mothers were cultured for 96 hr total. Late life DR (AL to DR) is necessary and sufficient for production of longer L1 larvae (p_int_ = 0.0014, 2-way ANOVA, n = 4). Mean and SEM are plotted for A,B,E,F,H.

Progeny size was increased in both DR models. *eat-2(ad465)* embryos were 5% longer than wild-type (WT) (Fig 2A). Diluting *E*. *coli* HT115 or HB101 in liquid culture increased progeny embryo length 4% (Fig 2B and 2C and S2A Fig). These relatively modest increases in embryo length are more striking in light of decreased maternal size (Fig 1A–1F). Embryonic development is constrained by the egg case, and though embryogenesis includes elongation, the size and shape of the embryo does not change. Indeed, increased embryo length translated into increased L1-stage larval length (during arrest, before growth commences) across a range of bacterial densities (Fig 2C and S2B Fig). We defined 25 mg/mL HB101 as *ad libitum* (AL) and 3.1 mg/mL (an eight-fold dilution) as DR. DR significantly increased progeny embryo length but width was not affected (Fig 2D). Embryos from AL mothers had an average aspect ratio (width/length) of 0.641 compared to 0.616 for DR progeny (p = 0.0003). Nevertheless, cross-sectional area of embryos was significantly increased (Fig 2E), indicating that increased embryo length affects overall embryo size. Taken together these results indicate that progeny size can be evaluated as embryo or L1 larval length. Comparable differences in progeny size using DR in liquid culture with HB101 or HT115 dilution and an *eat-2* mutant on solid media with OP50 demonstrates that the response to nutrient availability is not sensitive to *E*. *coli* strain or DR model.

Because DR delays development (by approximately 14% based on Fig 1I), differences in developmental age between chronologically synchronous populations of AL and DR worms could account for differences in progeny size. We measured progeny L1 length throughout early adulthood to address this possibility. Progeny L1 length increased between 72 and 96 hr in culture, indicating that maternal age influenced progeny size (Fig 2F). However, DR mothers consistently produced larger progeny than AL mothers. Increasing, rather than decreasing, progeny size with maternal age during early adulthood demonstrates that developmental delay does not explain the effect of DR on progeny size. Nonetheless, it is interesting to discover an additional influence on progeny size, further supporting the conclusion that progeny size is subject to variation.

We wondered when nutrient availability is assessed. Perturbations of larval development can affect reproduction and lifespan [36–39], suggesting a physiological set point is established that affects reproduction independent of conditions experienced during adulthood. Alternatively, nutrient availability could affect progeny size relatively late in development (during oogenesis), suggesting current nutrient status is assessed. To address this question, we transferred worms from AL to DR and DR to AL every 24 hr during 96 hr of culture (Fig 2G). Worms that experienced DR later in development had longer L1 progeny than worms that experienced DR earlier (Fig 2H). Exposure to AL or DR for only the last 24 hr in culture (young adulthood) caused production of relatively short or long L1 progeny, respectively. These data suggest nutrient status is sensed in real time, with a relatively immediate effect on oocyte provisioning.

### Maternal insulin-like signaling in the soma regulates progeny size

**Fig 3:**
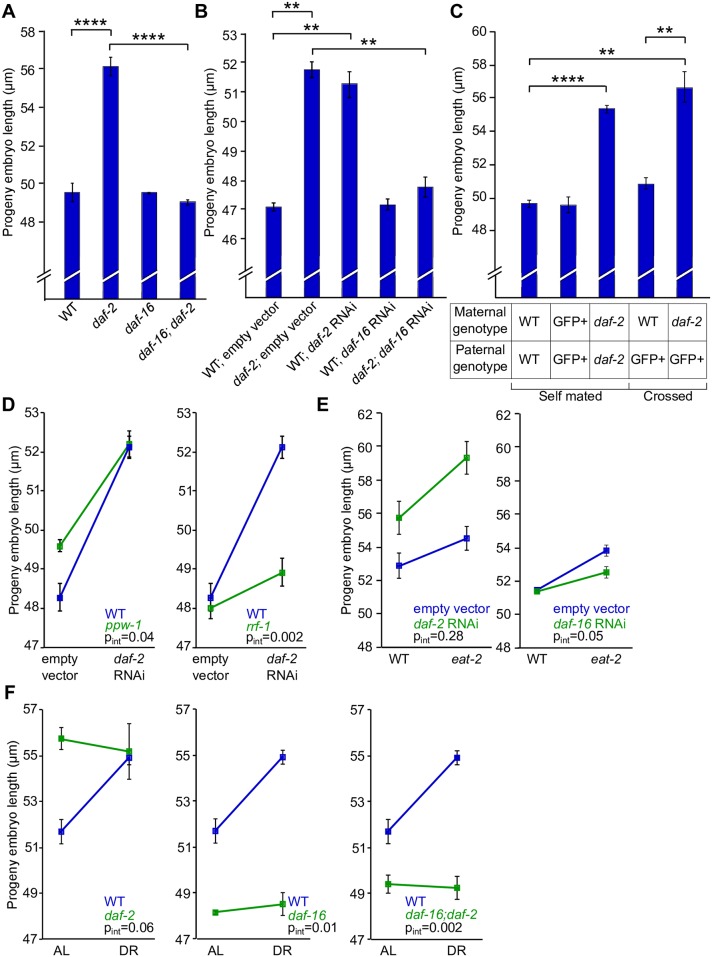
Maternal insulin-like signaling mediates the effect of diet in liquid culture on progeny size. A-B) Progeny embryo length for a variety of genotypes and RNAi treatments is plotted. C) Progeny embryo length for self and cross progeny resulting from crossing WT GFP+ males with WT and *daf-2* hermaphrodites is plotted. A-C) **p<0.01, ****p<0.0001, paired t-test, n = 3. D-F) Progeny embryo length is plotted for *daf-2* RNAi in *ppw-1* and *rrf-1* backgrounds (D), *daf-2* and *daf-16* RNAi in the *eat-2* DR system (E) and *daf-2*, *daf-16*, and double mutants in the liquid culture DR system (F). p_int_ indicates the p-value for the interaction term from a 2-way ANOVA for the two strains plotted. Mean and SEM are plotted for A-F. Note the expanded scale of the y-axis in E.

Reduction of insulin-like signaling increases progeny size, similar to DR. The sole *C*. *elegans* insulin-like receptor *daf-2*/InsR signals through the phosphoinositide 3-kinase signaling pathway to antagonize the forkhead box O transcription factor *daf-16/*FoxO, excluding it from the nucleus [40,41]. When cultured on plates (AL conditions), *daf-2*/InsR mutants and *daf-2* RNAi phenocopy DR with respect to embryo length and cross-sectional area without affecting adult body size (Fig 3A and 3B and S3 Fig). *daf-16*/FoxO mutant embryo length and area are comparable to WT on plates. Mutation of *daf-16* and *daf-16* RNAi fully suppressed the *daf-2* mutant large-embryo phenotype. Thus, *daf-16*/FoxO is epistatic to *daf-2*/InsR, suggesting the canonical insulin-like signaling pathway regulates progeny size. To isolate maternal effects of reduced insulin-like signaling we crossed WT males (carrying a fluorescent GFP reporter) into WT and *daf-2* mutant hermaphrodites (Fig 3C). Homozygous *daf-2* mutant mothers produced heterozygous cross progeny that were longer as embryos than homozygous cross progeny from WT mothers (p = 0.009). Thus, reduced maternal insulin-like signaling is sufficient to induce production of larger embryos.

Insulin-like signaling can function in a variety of anatomical sites, including the soma and germline, depending on the phenotype assayed [42–45]. *ppw-1* and *rrf-1* mutants are generally deficient for germline and somatic RNAi, respectively [46,47]. Somatic *daf-2* knockdown in the *ppw-1* mutant is sufficient for production of large embryos (p = 0.02), but germline *daf-2* knockdown in the *rrf-1* mutant did not significantly affect embryo size (Fig 3D). The *rrf-1* mutant actually maintains some capacity for RNAi in the soma [48], and lack of effect of *daf-2* RNAi in the mutant suggests somatic *daf-2* knockdown is not at a sufficient level or in the appropriate location to drive changes in progeny size. These data suggest that insulin-like signaling in the soma but not the germline regulates progeny size.

### Insulin-like signaling is required for progeny-size plasticity in response to food dilution

We wondered if insulin-like signaling mediates progeny-size plasticity in response to nutrient availability or if it affects progeny size independently. We used a paired t-test to compare L1 size between conditions in each mutant, with a significant p-value suggesting an effect of diet on the strain (plasticity). However, a non-significant t-test does not imply no effect of diet on length. We also used a two-way ANOVA to test each mutant for a difference from WT with respect to progeny-size plasticity, interpreting a significant interaction term (p_int_) as evidence that a particular strain exhibits a different reaction norm than WT. *daf-2* RNAi in the *eat-2* background produces exceptionally large embryos (Fig 3E). *eat-2(ad465); daf-2(RNAi)* embryos are significantly larger than *eat-2* embryos fed empty vector and WT embryos fed *daf-2* RNAi (p = 0.002 and p = 0.005, respectively), revealing additivity of *eat-2(ad465)* and *daf-2* RNAi. *daf-16* RNAi suppressed plasticity in the *eat-2* system (p_int_ = 0.05), but suppression was incomplete in that there was a modest residual effect of the *eat-2* mutant with *daf-16* RNAi (p = 0.04; Fig 3E). Together these results suggest that *eat-2* and insulin-like signaling affect progeny size independently.

Though *eat-2* is a popular genetic model for DR, different *eat* mutants are pleiotropic and affect more than pharyngeal pumping [33]. In contrast to the *eat-2* system, *daf-2*/InsR mutants did not display progeny-size plasticity in response to food dilution in liquid, and DR did not enhance the *daf-2* large-embryo phenotype (Fig 3F). These results suggest that insulin-like signaling mediates the effect of diet on progeny size. *daf-16*/FoxO mutants also did not display plasticity with food dilution, with mutant embryos being smaller than WT in AL and DR (Fig 3F). The *daf-16; daf-2* double mutant embryos also did not display plasticity and were smaller than WT in AL and DR, confirming that *daf-16* is epistatic to *daf-2* in liquid culture as on plates (Fig 3A and 3B and S3A and S3B Fig). These results further support the conclusion that nutrient availability acts through insulin-like signaling to affect progeny size, despite its relative lack of importance in the *eat-2* system.

### *skn-1*/Nrf, *pha-4*/FoxA and *nhr-49*/Hnf4 are required for progeny-size plasticity

**Fig 4:**
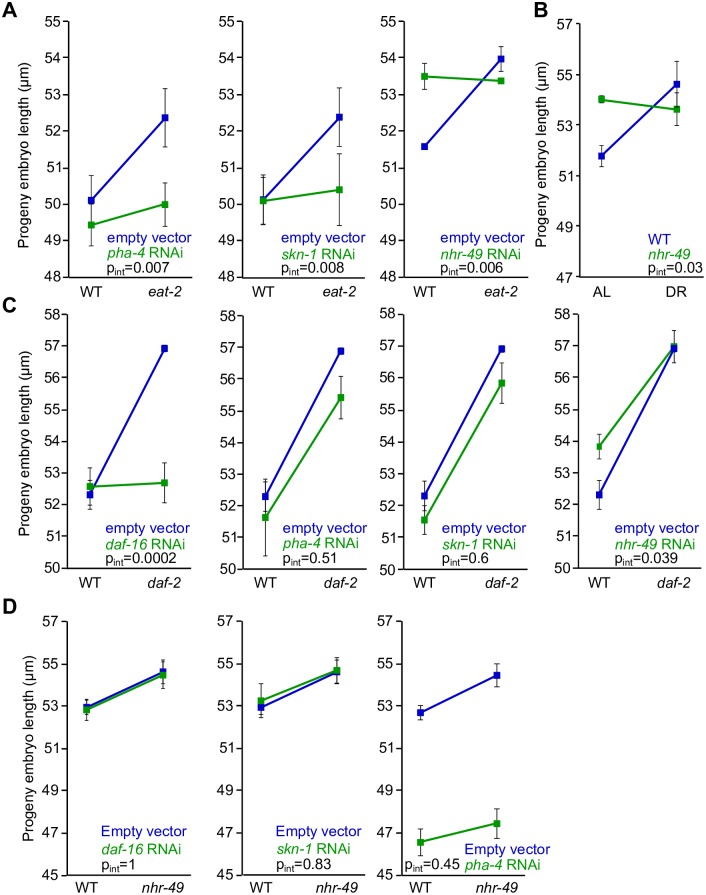
*pha-4*/FoxA, *skn-1*/Nrf, and *nhr-49*/Hnf4 are required for progeny-size plasticity. A-D) Progeny embryo length is plotted for RNAi of *pha-4*, *skn-1*, and *nhr-49* in the *eat-2* system (A), *nhr-49(nr2041)* in the food dilution system (B), RNAi of *daf-16*, *pha-4*, *skn-1*, and *nhr-49* in *daf-2(e1370)* (C), and RNAi of *daf-16*, *skn-1*, and *pha-4* in *nhr-49(nr2041)* (D). Mean and SEM are plotted. p_int_ indicates the p-value for the interaction term from a 2-way ANOVA for the two strains plotted.

Other genes known to regulate responses to nutrient stress are required in addition to insulin-like signaling for progeny-size plasticity. RNAi of *pha-4*/FoxA and *skn-1*/Nrf abolished plasticity in the *eat-2* system, causing *eat-2* embryos to be shorter (p = 0.007 and p = 0.02, respectively; Fig 4A). RNAi of *nhr-49* also abolished plasticity in the *eat-2* system, causing WT embryos to be longer (p = 0.04). Likewise, *nhr-49*/Hnf4 mutants did not display plasticity with food dilution, with larger mutant embryos than WT in AL (p = 0.02; Fig 4B). In AL conditions on plates, *nhr-49* mutants produced longer embryos and worms overexpressing *nhr-49* had shorter embryos (p = 0.03, p = 0.01, S4A Fig). These changes in embryo length were independent of adult length (S4B Fig). These results suggest that *pha-4*/FoxA, *skn-1*/Nrf and *nhr-49*/Hnf4 are each required for progeny-size plasticity in response to nutrient availability, with *nhr-49* activity limiting size and *pha-4* and *skn-1* activity increasing size.

We wondered if insulin-like signaling acts through or is affected by *pha-4*/FoxA, *skn-1*/Nrf, or *nhr-49*/Hnf4 to regulate progeny size. We exposed *daf-2* mutants, which have constitutively large embryos, to *pha-4*, *skn-1*, *nhr-49*, or *daf-16* RNAi. *daf-16* RNAi suppressed the large size of *daf-2* embryos on plates (Fig 4C), as expected (Fig 3A and 3B and S3A and S3B Fig). *pha-4* and *skn-1* RNAi did not suppress the *daf-2* large-embryo phenotype. Lack of suppression suggests that *pha-4* and *skn-1* function independently or upstream of insulin-like signaling. *nhr-49* RNAi increased embryo size in WT, as expected (Fig 4A and 4B), but it did not enhance the large-embryo phenotype of *daf-2* (Fig 4C). Lack of enhancement suggests *nhr-49* and *daf-2* function in the same pathway.

We extended epistasis analysis with an *nhr-49* mutant. *nhr-49* mutant embryos produced on plates were significantly larger than WT (p = 0.02; Fig 4D), as expected (Fig 4C and S4A Fig). *daf-16* RNAi did not suppress this large-embryo phenotype (p = 0.03; Fig 4D). Lack of suppression suggests *daf-16* does not act downstream of *nhr-49* to regulate embryo size. Together with the results of *nhr-49* RNAi in the *daf-2* background (Fig 4C), these results suggest that *nhr-49* functions downstream of insulin-like signaling. However, the effect of *nhr-49* on embryo size is smaller than that of *daf-2*, suggesting *daf-16* regulates additional effectors as well. *skn-1* RNAi also does not suppress the large-embryo phenotype of *nhr-49* mutants (p = 0.03), suggesting it does not function downstream of *nhr-49*. *pha-4* RNAi reduces embryo size in WT and *nhr-49*, but there is no interaction between genotype and RNAi, suggesting the effect of *nhr-49* on progeny size also does not depend on *pha-4* (Fig 4D). Together with the results of *skn-1* and *pha-4* RNAi in the *daf-2* background (Fig 4C), these results suggest that *skn-1* and *pha-4* function in parallel to insulin-like signaling and *nhr-49* to regulate progeny size.

### Dietary restriction buffers progeny from starvation

We wondered how persistent the maternal effects of DR are and if they are possibly adaptive. We did not observe a grandmaternal effect of nutrient availability on F_2_ progeny size (S5A Fig). That is, the effects of nutrient availability in a given generation override the effects from the previous generation. We also measured F_1_ progeny length after 48 hr of development on plates (AL conditions). There was no detectable difference in progeny length after 48 hr of development (S5B Fig), suggesting the differences in size observed for embryos and L1 larvae dissipate during postembryonic development in rich conditions. Likewise, there was also no effect on progeny adult lifespan (S5C Fig). These results suggest that the effects of maternal diet are transient and do not have a fitness consequence when progeny are cultured in rich conditions.

**Fig 5:**
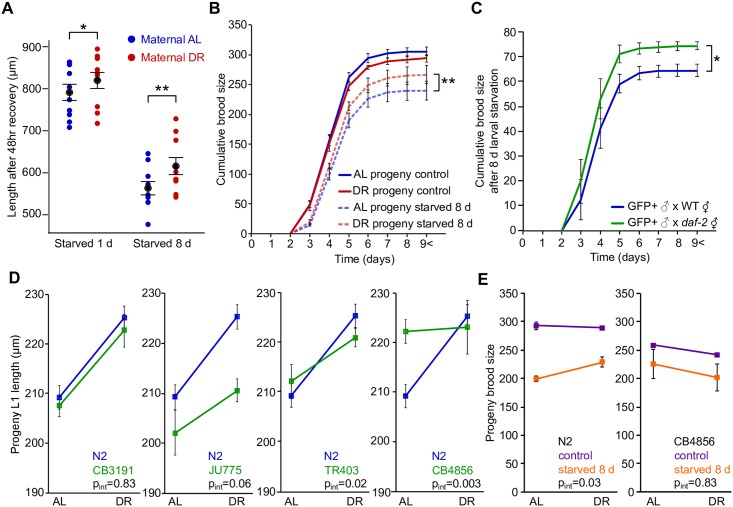
DR and reduced maternal insulin-like signaling buffer progeny against L1 starvation. A) Average length after 48 hr of postembryonic development of AL and DR progeny that were starved for 1 d or 8 d as L1 larvae is plotted (10 biological replicates with grand mean and SEM in black; *p = 0.05, **p = 0.008, paired t-test, n = 10). B) Cumulative brood size on plates is plotted for AL and DR progeny that were not starved (control) or starved for 8 d as L1 larvae (**p = 0.001, unpaired t-test on total brood size, n = 3); C) Cumulative brood size is plotted for cross progeny that were starved for 8 d as L1 larvae. GFP+ males were mated with WT (blue) or *daf-2* hermaphrodites (green) (*p = 0.03, unpaired t-test, n = 3). D) Natural isolates demonstrate variability in progeny plasticity. L1 length is plotted for AL and DR progeny of N2 and four wild isolates. N2 L1 length increases in DR (p = 1.6x10^-6^, paired t-test, n = 15). p_int_ indicates the p-value for the interaction term from a 2-way ANOVA for the two strains plotted. E) Brood size is plotted for AL and DR progeny of N2 and CB4856 that were not starved (control) or starved for 8 d. The 2-way ANOVA interaction term p-value is reported for each strain (n = 3). Mean and SEM of biological replicates are plotted in B-E.

We wondered if DR progeny have a potential fitness advantage in conditions of nutrient stress. We previously showed that extended L1 starvation subsequently delays growth and reduces fertility [49]. Progeny of AL and DR worms showed no difference in L1 starvation survival when survival is scored as the ability to move to food (S5D Fig). However, this assay is not as sensitive as measuring growth or reproduction after recovery from starvation. We therefore starved AL and DR progeny for 8 d as L1 larvae, then cultured them on plates and measured growth and fertility. Animals that experienced 8 d L1 starvation were 29% shorter after 48 hr postembryonic development and had 21% fewer progeny than controls (Fig 5A and 5B), confirming the effects of extended L1 starvation [49]. After 1 d of L1 starvation and 48 hr of postembryonic development (1 d of starvation was used for synchronization), DR progeny were 4% longer than AL (p = 0.05; Fig 5A). After 8 d of starvation, DR progeny were 9% longer than AL (p = 0.008). Furthermore, after 8 d of starvation, DR progeny produced 11% more offspring than AL (p = 0.001; Fig 5B). Two-way ANOVA revealed a significant interaction between maternal diet (AL or DR) and progeny starvation (unstarved control or starved 8 d) on progeny brood size (p_int_ = 0.0068). These results suggest a fitness advantage to progeny of DR worms when they hatch in the absence of food.

Having shown that insulin-like signaling mediates effects of maternal diet on progeny size (Fig 3), and that increased size is correlated with a fitness advantage when progeny encounter L1 starvation (Fig 5B), we wondered if maternal insulin-like signaling regulates the ability of progeny to resist starvation. Using genetic crosses, we found that after 8 d of L1 starvation heterozygous cross-progeny from homozygous *daf-2* mutant mothers produced 15% more progeny than homozygous cross-progeny from WT mothers (p = 0.03, Fig 5C). Brood sizes are smaller in Fig 5C than Fig 5B due to the GFP reporter used to mark cross progeny, but this effect is controlled for. These results support the conclusion that maternal diet acts through insulin-like signaling in the mother to buffer progeny from the reproductive costs of L1 starvation, contributing to a fitness advantage in conditions of nutrient stress.

### Natural variation of progeny-size plasticity

We wondered if wild isolates of *C*. *elegans* display progeny-size plasticity similar to the WT reference strain (Bristol N2), so we cultured wild isolates in AL and DR conditions and measured progeny L1 length. We found that CB3191 displayed significant plasticity (p = 0.006) comparable to N2 (p_int_ = 0.83) (Fig 5D). JU775 and TR403 displayed significant plasticity as well (p = 0.03 and p = 0.04, respectively), but not to the same extent as N2 (p_int_ = 0.06 and p_int_ = 0.02, respectively). The Hawaiian strain CB4856 did not exhibit plasticity (p = 0.81), responding significantly differently than N2 to DR (p_int_ = 0.003). Observation of progeny-size plasticity among wild strains suggests it is not a domestication artifact of N2, as seen for other phenotypes [50].

Because the Hawaiian strain, CB4856, did not display progeny-size plasticity, we tested whether maternal DR buffers progeny from the reproductive costs of L1 starvation in this strain. As expected (Fig 5B), we reproduced the effect of maternal diet on progeny brood size following 8 d L1 starvation in N2, with a significant interaction between maternal diet (AL or DR) and progeny starvation (unstarved control or starved 8 d) on progeny brood size (p_int_ = 0.03; Fig 5E). However, as for progeny size, maternal diet did not affect progeny fertility after 8 d L1 starvation in CB4856, with AL and DR progeny displaying a similar reproductive cost following 8 d starvation (p_int_ = 0.83). These results show that natural variation of intergenerational plasticity affects progeny size and starvation resistance, extending the correlation between these two traits from N2 and *daf-2* mutants to CB4856. Our analysis of wild isolates suggests that genetic differences among strains modify their response to nutrient stress, affecting the reaction norm of intergenerational plasticity.

## Discussion

**Fig 6:**
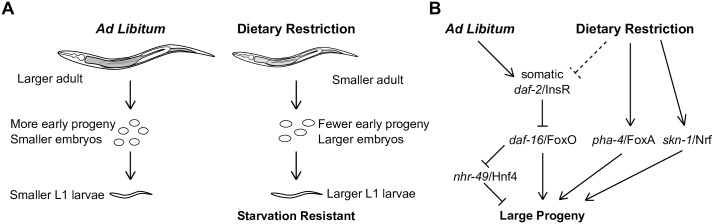
Model for the impact of maternal diet on progeny size and starvation resistance. A) Mothers that experience conditions of dietary restriction are smaller and produce fewer but larger embryos and L1 progeny. Progeny of DR mothers are resistant to developmental delay and reduced brood size after recovery from extended L1 starvation. B) *daf-2*/InsR acts through *daf-16*/FoxO in the soma to regulate embryo size. *nhr-49* is repressed by *daf-16* in DR, and *nhr-49* functions in AL to reduce progeny size. *skn-1* and *pha-4* are active in DR and function in parallel to insulin-like signaling and *nhr-49* to increase progeny size. Insulin-like signaling is required to increase progeny size in food dilution DR but not the *eat-2* genetic model (dashed line).

It is well established that an individual's diet affects their phenotype. In humans, maternal diet also influences progeny phenotype [51]. Given a shared blood supply, maternal effects of diet are not surprising in placental mammals. However, in invertebrates embryonic development is relatively independent of the mother and uterine environment after fertilization. Nonetheless, we show in *C*. *elegans* that maternal diet affects progeny as well, resulting in intergenerational phenotypic plasticity (Fig 6A). Though DR worms are smaller and produce fewer progeny, they produce larger progeny. Effects on progeny size are relatively modest but robust in that size is affected by two different models of DR with liquid and solid media, with three different food sources, and in some wild isolates. Maternal age and various mutants also affect progeny size, further demonstrating plasticity in this trait. Moreover, progeny of DR mothers are buffered from starvation in the first larval stage, with increased growth and fertility compared to AL progeny following starvation, and we show that maternal insulin-like signaling controls this apparent fitness advantage. Larval starvation is likely common in the wild and preceded by limited nutrient availability to the mother, suggesting intergenerational plasticity is adaptive.

### Maternal insulin-like signaling regulates intergenerational plasticity

We provide genetic evidence that somatic maternal insulin-like signaling regulates oocyte provisioning in response to nutrient availability. Maternal effects of nutrient availability have been described for *C*. *elegans* and *Drosophila*, but nothing was known about their regulation [14,37–39]. While studies in *Drosophila* have focused on parental larval diet [37–39], we show that limited food during adulthood, but not larval development, alters provisioning to similarly produce larger offspring. This suggests that diverse invertebrates have qualitatively similar but developmentally distinct responses to nutrient availability. We used genetic crosses to demonstrate a maternal effect of insulin-like signaling on progeny size, supporting the conclusion that insulin-like signaling regulates oocyte provisioning. We also used genetic crosses to demonstrate that the effects of maternal insulin-like signaling extend beyond progeny size to regulate the ability of progeny to retain fertility after extended L1 starvation. Germline- and soma-specific RNAi of *daf-2*/InsR suggest that insulin-like signaling functions somatically to regulate progeny size. Somatic site of action suggests nonautonomous function, implying that insulin-like signaling regulates soma-to-germline signaling, though the nature of such a signaling pathway is unclear.

Insulin-like signaling mediates progeny-size plasticity in an explicit model of DR involving dilution of food in liquid culture. That is, mutations affecting *daf-2*/InsR and *daf-16*/FoxO abolished plasticity, rendering progeny constitutively large and small, respectively. Creating DR conditions by food dilution is well controlled and free of confounding effects. Nonetheless, we complemented this DR model using the popular *eat-2* genetic model of DR. *eat-2* encodes a nicotinic acetylcholine receptor subunit that affects pharyngeal pumping [33,52], and there are caveats that suggest cautious interpretation of *eat-2* results. For example, *eat-2* mutants are possibly pleiotropic [23,33], and in contrast to food dilution, which is conditional, mutant animals have experienced DR continuously for many generations, possibly confounding immediate and epigenetic effects of DR. Though *eat-2* mutants produced large progeny as with food dilution, *daf-2* RNAi and *eat-2* mutation had additive effects on progeny size, and *daf-16* mutation did not completely abolish plasticity in the *eat-2* system. These results suggest that DR in the *eat-2* system does not act through insulin-like signaling. The discrepancy in the relative importance of insulin-like signaling in these two DR models is not surprising given caveats of the *eat-2* system and results of aging studies, which show that the importance of insulin-like signaling to lifespan extension by DR depends on the DR model [27]. In particular, *daf-16*/FoxO null mutation completely abolishes lifespan extension by food dilution but not *eat-2* mutation (*daf-2*/InsR mutants are not null and therefore difficult to interpret) [23,26,27], consistent with our results for progeny size. In any case, our results clearly demonstrate that directly manipulating nutrient availability (food dilution) acts through insulin-like signaling to alter maternal provisioning.

### A regulatory network that controls aging also regulates progeny size

Homeostatic regulators *pha-4*/FoxA, *skn-1*/Nrf, and *nhr-49*/Hnf4 are each required for progeny-size plasticity (Fig 6B). Disruption of *pha-4* or *skn-1* suppressed the *eat-2* large-embryo phenotype, suggesting that they function in this DR model to increase progeny size. Such a role is analogous to their role in aging in that they are required for DR to increase lifespan in the *eat-2* system [28,29]. The relatively small size of embryos resulting from disruption of *pha-4* or *skn-1* suggests that they promote increased size in DR, analogous to their activity increasing lifespan in DR. Disruption of *nhr-49* abolished progeny-size plasticity in the food dilution and *eat-2* DR systems. *nhr-49* loss of function increased progeny size and overexpression decreased progeny size, suggesting that, in contrast to *pha-4* and *skn-1*, *nhr-49* limits progeny size in AL.

Genetic epistasis analysis suggests that the regulatory network comprised of insulin-like signaling, *pha-4*/FoxA, *skn-1*/Nrf, and *nhr-49*/Hnf4 that regulates lifespan also controls maternal provisioning and progeny size. Our results suggest that *nhr-49* functions downstream of insulin-like signaling. Likewise, *nhr-49* functions downstream of *daf-16* to delay aging in germ cell-ablated worms [32]. Our results suggest that *pha-4* and *skn-1* function in parallel to insulin-like signaling and *nhr-49*. Consistent with our results, *pha-4* and *skn-1* are thought to regulate aging in parallel to insulin-like signaling [27], though their relationship to *nhr-49* in the context of aging has not been addressed. Together the results of our genetic analysis and published work on aging portray a common regulatory network mediating effects of nutrient availability on lifespan, maternal provisioning and progeny size.

Our work raises the very interesting question of how this regulatory network translates maternal nutrient availability into effects on progeny size and the ability to cope with nutrient stress. Reduced insulin-like signaling increases fat stores [53,54], suggesting a role for fat. In addition, DR embryos have relatively large lipid droplets [55], suggesting altered provisioning. *nhr-49* and *skn-1* regulate the distribution of fat between the germline and soma. *nhr-49* controls oxidation and desaturation of fat in response to germline cues [30,32], and fat allocation shifts from soma to germline in *eat-2* mutants and *skn-1* gain-of-function adults [56]. We speculate that DR increases relative allocation of fat to the germline, bolstering provisioning and buffering progeny from nutrient stress.

### Progeny quality and starvation resistance

Reproduction is typically thought to occur at the expense of somatic maintenance and lifespan [18,57]. Using fecundity as a proxy for reproductive output simplifies analysis of this putative trade-off, but it does not fully reflect biological complexity. Our findings suggest an additional trade-off between progeny quantity and quality in that DR mothers produce fewer but larger progeny that cope better with early-life starvation. These observations demonstrate the importance of progeny quality as an additional metric for reproductive fitness [57].

Our work raises the question of what it means to be starvation resistant. We assayed three different adverse effects of L1 starvation: death or other loss of mobility, growth rate during recovery, and fertility. Maternal DR affected the last two of these effects but not the first. It is tempting to equate starvation resistance with survival, assayed by movement, but from a physiological, ecological and evolutionary perspective resistance should be evaluated in terms of recovery and reproductive fitness. Indeed, larvae lose the ability to recover from starvation before they lose the ability to move. Furthermore, mutants have been identified that dramatically compromise the ability to recover from L1 starvation though survival itself is not affected [58]. By assaying adverse effects of L1 starvation in these different ways we were able to identify a biologically significant effect of maternal DR on starvation resistance that we would have missed if we considered resistance only in terms of immediate survival.

### Evolutionary significance of intergenerational plasticity

Fitness consequences of maternal effects hinge on anticipation of conditions. The "thrifty phenotype" hypothesis proposes that children of mothers malnourished during pregnancy are programmed to store more energy in anticipation of limited nutrient availability [8,59,60]. When challenged with starvation upon hatching, DR progeny subsequently grew faster and had more offspring than AL progeny. We did not find a putative fitness effect in DR progeny grown in AL without experiencing starvation. Thus, we observe a similar contingency as for the thrifty phenotype: fitness consequences of maternal diet depend on environmental conditions experienced by progeny. Theory and *in vitro* evolution of *C*. *elegans* suggest that anticipatory maternal effects underlie adaptation to regularly alternating environments [61]. We speculate that eggs produced during DR regularly hatch in the absence of food, as a result of populations experiencing resource limitation prior starvation. Furthermore, we show using genetic crosses that insulin-like signaling in the mother regulates the ability of her progeny to cope with nutrient stress. These results suggest that maternal insulin-like signaling controls metabolic programming and anticipation of environmental conditions in progeny. We propose that this intergenerational and potentially adaptive function of insulin-like signaling is conserved in other animals.

We found the reaction norm for intergenerational plasticity varies among wild strains, with some displaying no plasticity at all. The ecology of *C*. *elegans* is coming to light but remains poorly understood in that we do not understand the dynamics of nutrient conditions or how they vary for different populations [62]. Natural variation in intergenerational plasticity suggests existence of genetic variants that influence the trait, which will be interesting to identify. It is intriguing to speculate that such natural variation reflects evolutionary adaptation to different niches, but future work is necessary to determine the causes and significance of such phenotypic variation.

## Materials and Methods

### Liquid culture system for dietary restriction

HT115 and HB101 strains of *E*. *coli* were grown in LB with streptomycin in 5 mL starter cultures used to inoculate 0.5 L cultures in TB. Starter cultures were grown for ~6 hr at 37°C and the larger 0.5 L cultures were grown overnight (approximately 16–20 hr) with shaking. Bacteria were pelleted by centrifugation and resuspended at 250 mg/mL in S-complete buffer to prepare 10x stocks, and these stocks were stored at 4°C and used within several weeks. The 10x stocks of bacteria were diluted in S-complete for liquid culture experiments [63]. HB101 is considered the standard bacteria for use in liquid culture, as OP50 is on plates. OP50 is known to clump in liquid. Based on optimization of embryo and L1 length differences from a dilution series (Fig 1C), AL was defined as 25 mg/mL (determined to be 7.5x10^8^ cfu) HB101 and DR was defined as 3.1 mg/mL (determined to be 8.75x10^7^ cfu), an eight-fold dilution of AL.

Progeny size was determined for a dilution series of *E*. *coli* HT115 (Fig 2B), but HB101 was used in all other instances. HT115 was prepared with the same protocol as HB101. Optical density at 600 nm (OD600) was measured with a spectrophotometer. Initial measurement confirmed that over time bacteria do not grow in S-complete culture (S1 Fig). Subsequently OD600 was measured in 1:4 dilutions to ensure measurements were in the linear range of measurements. Absorbance readings were consistently around 0.7–0.8 for AL and 0.09–0.1 for DR (S1 Fig).

Worms fed *E*. *coli* OP50 on NGM plates were bleached and progeny were arrested as L1 larvae by hatching in virgin S-basal buffer (no ethanol or cholesterol). L1 larvae were added to liquid cultures at a density of 1 worm/100 μL (S1A Fig). At this density, worms did not consume enough bacteria in culture over the 96 hr culture period to significantly alter the density of bacteria (S1C Fig). This density is comparable but slightly reduced from the 15 worms/mL density used in a similar system for DR in liquid culture [34]. Worms were cultured in Erlenmeyer flasks at 20°C with shaking at 180 rpm. After 96 hr in culture, worms were collected by centrifugation and embryos (progeny) were harvested by standard hypochlorite treatment. Because they were grown in liquid, eggs that had been laid but not hatched survived hypochlorite treatment and were included in the progeny cohort. This is different from collecting worms from plates for hypochlorite treatment, where eggs stick to the solid media and only those that are *in utero* are harvested.

### Culture on solid media

Standard techniques for culture on plates with nematode growth media (NGM) was used for the genetic model of DR (*eat-2* mutant vs. WT), all RNAi experiments, and phenotypic analysis of progeny. Stationary cultures of *E*. *coli* OP50 were used to seed NGM plates to produce a lawn prior to plating embryos or larvae, except for RNAi experiments, which used HT115 carrying different RNAi plasmids (see below). Plates were always incubated at 20°C.

### Reproductive onset and brood size

To measure reproductive onset (Fig 1I), worms were grown in AL and DR conditions. Worms were sampled at the indicated times. At each timepoint a 10 mL sample of the culture was centrifuged and nearly all of the supernatant was removed. The remaining volume was plated onto unseeded NGM plates. Worms were scored with a stereoscope to determine if they were carrying fertilized embryos. Worms with at least one fertilized embryo *in utero* were considered reproductive.

Early fecundity of P0 worms was measured after 96 hr in liquid culture in slightly modified AL and DR conditions (Fig 1J). To measure brood size of individual animals, single L1 worms were added to 1 mL cultures of AL (25 mg/mL HB101) or DR (3.1 mg/mL HB101) in 12 mL round bottom polystyrene tubes at 20°C with shaking at 220 rpm. After 96 hr of growth, each culture was diluted with ~5 mL virgin S-basal and centrifuged at 3000 rpm for 1 min. Nearly all supernatant was removed, the remaining volume was plated on an unseeded 6 cm NGM plate, and the number of progeny present was counted with a stereoscope.

To determine progeny brood size (Fig 5B and 5E), progeny were harvested from worms grown in AL or DR conditions by hypochlorite treatment after 96 hr in culture. Progeny were either plated immediately after hypochlorite treatment as embryos (control) or as arrested L1 larvae (starved 8 d) onto NGM plates seeded with OP50. To isolate embryos from crosses (Fig 5C) mothers were transferred to fresh plate at 8-12hr intervals and embryos were washed from plates. Arrested larvae were prepared by culturing embryos from hypochlorite treatment or embryo harvest in virgin S-basal at 1 worm/μL for 8 d. After 48 hr on plates, 25 worms per condition were singled onto 6 cm NGM plates seeded with a small lawn of OP50. Worms were transferred to fresh plates at 24 hr intervals, and the number of offspring was counted 2 days after removal of the mother.

### Measuring embryo and larval size

Progeny were collected by hypochlorite treatment of worms after 96 hr of culture. Embryo length measurements are constant during development due to the rigid eggshell, so developmental stage is not a confounding factor. To measure embryo size, embryos were plated onto unseeded 10 cm plates with NGM agar. These were imaged with a Zeiss Discovery.V20 stereomicroscope with a 10x objective (KSC 190–975). The images were analyzed using FIJI and calibrated to a micrometer. Lengths of embryos were measured both manually and by automatically thresholding embryos and calculating the long axis from ellipse fitting. Area and width measurements were also generated by thresholding. Specifically, background was subtracted, images were thresholded, they were converted to binary, holes were filled, and then particles were analyzed. This analysis was done in batch and the results were manually curated to ensure only quality embryo images were used.

Size of L1 larvae and worms cultured for 48 or 96 hr (starting from L1 arrest) was measured using the Wormsizer software [64]. Worms were washed with S-basal and plated on unseeded 10 cm NGM plates. These were imaged with a Zeiss Discovery.V20 stereomicroscope and magnification was adjusted based on the stage of the worms. Representative images of adult worms were taken at 10x on a Zeiss AxioImager compound microscope.

### RNA interference

RNAi bacteria (HT115) were grown at 37°C in starter cultures of LB with tetracycline. Starter cultures were used to innoculate 0.5 L TB with tetracycline and these large cultures were incubated overnight (approximately 16–20 hr). Bacteria were concentrated to 250 mg/mL and frozen in S-complete with 15% glycerol at -80°C. Bacteria were thawed and plated on NGM with 25 μg/mL carbenicilin and 1 mM IPTG. Bacterial clones from the OpenBio RNAi library were used. RNAi plasmids were confirmed by sequencing.

### Crosses

The SJ4103 mitochondrial GFP reporter strain was used as a source of males to mark cross progeny. Males were initially generated by heat shocking worms and backcrossing was used to expand and maintain the population. Males were mated to WT or *daf-2* hermaphrodites and only fluorescent cross progeny were selected for analysis in Figs 3C and 5C.

### Lifespan

To measure progeny lifespan (S5C Fig), progeny of AL and DR worms were harvested by hypochlorite treatment after 96 hr in culture. Progeny were picked as young adults onto fresh plates and moved every other day to a new plate to isolate them from their offspring. Six plates of 20 worms per condition were set up at the beginning of each experiment and plates were blinded. Survival was scored over time and worms that disappeared were censored. Three independent biological replicates were consistent, so data were pooled to generate a log-rank p-value using the OASIS program basic survival analysis [65].

### Starvation Survival

To measure starvation survival (S5D Fig), embryos were collected with hypochlorite treatment of AL and DR cultures and embryos were suspended at a density of 1/μL in 10 mL virgin S-basal in 25 mm glass test tubes on a tissue culture roller drum at 21–22°C. Arrested L1 worms were plated onto 6 cm plates beside the OP50 lawn, and the total number of worms plated (T_p_) was counted. Two days later the total number of worms alive on the plate was scored (T_A_). Survival was defined as T_A_/T_p_ at each timepoint. Survival curves and statistics were calculated as previously described [66]. Briefly, curves were fit to each biological replicate, median survival was determined, and an unpaired t-test was used to contrast average median survival between groups.

### Strains

The Bristol N2 strain was used and is annotated as wild-type (WT). The following strains and mutant alleles were also used: AGP24f glmEx5[P*nhr-49*::*nhr-49*::GFP + Pmyo-2::mCherry], CB1370 *daf-2(e1370)*, CB3191, CB4856, DA465 *eat-2(ad465)*, GR1309 *daf-16(mgDf47);daf-2(e1370)*, JU775, NL2098 *rrf-1(pk1417)*, NL2550 *ppw-1(pk2505)*, PS5150 *daf-16(mgDf47)*, SJ4103 zcIs14[myo-3::GFP(mit)], STE68 *nhr-49(nr2041)*, and TR403.

### Statistical Analysis

Unpaired t-tests were used for analysis of starvation survival (S5D Fig) and total brood size (Fig 5B and 5C). Paired t-tests were used for image-based analysis of progeny size to account for systematic variation in conditions from trial to trial. Each reference to n indicates the number of independent biological replicates. Such replicates used independent cultures prepared and assayed on different days.

## Supporting Information

S1 Fig A liquid culture system for DR. A) Schematic of DR by food dilution in liquid culture. Worms are grown in standard conditions on plates with OP50 and then bleached to obtain embryos. Embryos are hatched in buffer so they enter L1 arrest for synchronization. Arrested L1 larvae are added to culture flasks at a very low density of 10 worms/mL so that they do not reduce bacterial density during culture. *E*. *coli* HB101 is used for liquid culture to avoid flocculation. Worms are cultured at 20°C with shaking and typically harvested at 96 hr to collect their embryos for phenotypic analysis. B) Optical density at 600 nm (OD600) is plotted for different densities of HB101 over time in S-complete, showing that density is roughly constant. C) OD600 is plotted of 1:4 dilutions of AL and DR cultures with worms at 0 and 96 hr of culture. There is not a significant change in bacterial density in either AL or DR (p = 0.10, p = 0.19 respectively, paired t-test, n = 3). The data points obscure SEM bars.
(TIF)

S2 Fig Progeny of DR mothers are longer as embryos and L1 larvae. A) Embryo length increases with reduced maternal HB101 (*p = 0.01, 1-way ANOVA, n = 4). B) Progeny L1 length increases with reduced maternal HB101 (***p<0.0001, 1-way ANOVA, n = 6). Mean and SEM are plotted in A and B.
(TIF)

S3 Fig Reduced insulin-like signaling increases embryo area without altering adult size. A) Cross-sectional area of embryos is plotted for a variety of genotypes. *daf-2(e1370)* mutant embryos have significantly greater area than WT (p<0.001, paired t-test, n = 3) and *daf-16;daf-2* double mutants (p<0.001, paired t-test, n = 3). *daf-16(mgDf47)* single mutants and *daf-16;daf-2* double mutants are not significantly different from WT. B) Cross-sectional area of embryos is plotted for WT and *daf-2* mutants with and without RNAi of *daf-2* and *daf-16*. *daf-2* RNAi increases embryo area (p = 0.01, paired t-test, n = 3), consistent with the increased area of *daf-2* mutant embryos fed empty vector bacteria (p = 0.003, paired t-test, n = 3). *daf-16* RNAi in a *daf-2* mutant background suppresses the increase in embryo area (p<0.001, paired t-test, n = 3). C) Adult length is plotted for a variety of genotypes after 96 hr of culture (starting from L1 arrest). Length of WT worms is not significantly different from that of *daf-2* (p = 0.49, paired t-test, n = 3), *daf-16* (p = 0.24, paired t-test, n = 3), or *daf-16;daf-2* double mutants (p = 0.069, paired t-test, n = 3). Mean and SEM are plotted for A-C.
(TIF)

S4 Fig *nhr-49*/Hnf4 activity reduces embryo length independent of adult length. A) Overexpression (OE) of *nhr-49* reduces embryo length (p = 0.034, paired t-test, n = 4) and loss-of-function *nhr-49* mutation increases embryo length (p = 0.014, paired t-test, n = 4) in AL conditions on plates with OP50. B) Adult length at 96 hr is reduced in an *nhr-49* loss-of-function mutant (p = 0.02, paired t-test, n = 3) but not when *nhr-49* is overexpressed (p = 0.10, paired t-test, n = 3). Mean and SEM are plotted.
(TIF)

S5 Fig Maternal nutrient availability does not affect F_2_ embryo size, late larval size of progeny, progeny adult lifespan, or progeny L1 starvation survival. A) Size of embryos produced by progeny of AL or DR mothers is plotted for worms grown in AL and DR conditions. Diet of the F_1_ progeny, but not their mothers (P_0_) impacted embryo size (F_2_ generation) (p = 0.02 in both cases, paired t-test, n = 3) B) Length after 48 hr of postembryonic development (L4-stage larvae) is not significantly different for progeny of AL and DR worms that were not starved (p = 0.72, paired t-test, n = 10). C) Survival of adult worms on plates with OP50 is plotted over time. Progeny of DR worms do not have significantly altered lifespan (p = 0.58, log-rank test, 342 AL animals and 320 DR animals pooled from 3 biological replicates). Although statistics were done on pooled data the mean and SEM of 3 biological replicates are plotted. D) Survival of starved L1 larvae is plotted over time. Progeny of DR worms do not exhibit any difference in L1 starvation survival (p = 0.34, t-test on median survival, n = 4). Mean and SEM of 4 biological replicates are plotted.
(TIF)
