# Correction: Maternal Diet and Insulin-Like Signaling Control Intergenerational Plasticity of Progeny Size and Starvation Resistance

**DOI:** 10.1371/journal.pgen.1007639

**Published:** 2018-08-30

**Authors:** Jonathan D. Hibshman, Anthony Hung, L. Ryan Baugh

S1 Fig reports OD600 values for *ad libitum* (25 mg/mL *E*. *coli* HB101) and dietary restriction (3.1 mg/mL *E*. *coli* HB101) with incorrect dilution factors. The measurements reported in S1B Fig were from a 5-fold dilution and those in S1C were from a 20-fold dilution. The *ad libitum* and dietary restriction conditions corresponded to approximately 16 and 2 OD600 units, respectively. The authors are issuing a correction to rectify this mistake. Please view the corrected version of S1 Fig and its legend below.

These changes also affect two sentences in the Materials and Methods section.

**The fifth and sixth sentences in the second paragraph under the subheading “Liquid culture system for dietary restriction” in the Materials and Methods should read as follows**:

“Subsequently OD600 was measured in 1:20 dilutions to ensure measurements were in the linear range of measurements. At this dilution, absorbance readings were consistently around 0.7–0.8 for AL and 0.09–0.1 for DR (S1 Fig).”

## Supporting information

S1 Fig A liquid culture system for DR. A) Schematic of DR by food dilution in liquid culture. Worms are grown in standard conditions on plates with OP50 and then bleached to obtain embryos. Embryos are hatched in buffer so they enter L1 arrest for synchronization. Arrested L1 larvae are added to culture flasks at a very low density of 10 worms/mL so that they do not reduce bacterial density during culture. *E*. *coli* HB101 is used for liquid culture to avoid flocculation. Worms are cultured at 20°C with shaking and typically harvested at 96 hr to collect their embryos for phenotypic analysis. B) Optical density at 600 nm (OD600) is plotted for 1:5 dilutions of different densities of HB101 over time in S-complete, showing that density is roughly constant. C) OD600 is plotted for 1:20 dilutions of AL and DR cultures with worms at 0 and 96 hr of culture. There is not a significant change in bacterial density in either AL or DR (p = 0.10, p = 0.19 respectively, paired t-test, n = 3). The data points obscure SEM bars.
(TIF)
