# Supplementary figures and images for: Maternal Diet and Insulin-Like Signaling Control Intergenerational Plasticity of Progeny Size and Starvation Resistance
Source: PLoS Genet. 2016 Oct 26;12(10):e1006396. doi: 10.1371/journal.pgen.1006396 (PMC5081166; doi:10.1371/journal.pgen.1006396)

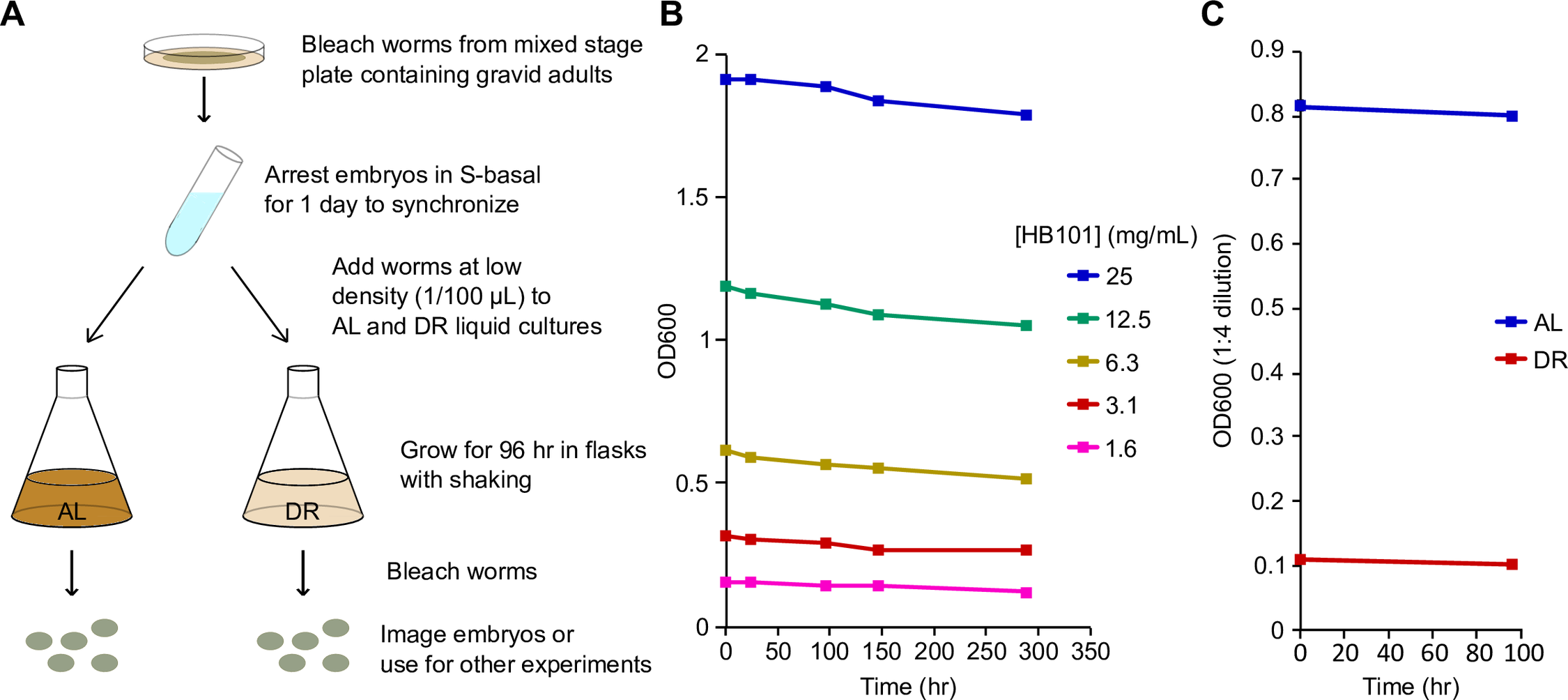

Supplement: S1 Fig — A) Schematic of DR by food dilution in liquid culture. Worms are grown in standard conditions on plates with OP50 and then bleached to obtain embryos. Embryos are hatched in buffer so they enter L1 arrest for synchronization. Arrested L1 larvae are added to culture flasks at a very low density of 10 worms/mL so that they do not reduce bacterial density during culture. E. coli HB101 is used for liquid culture to avoid flocculation. Worms are cultured at 20°C with shaking and typically harvested at 96 hr to collect their embryos for phenotypic analysis. B) Optical density at 600 nm (OD600) is plotted for different densities of HB101 over time in S-complete, showing that density is roughly constant. C) OD600 is plotted of 1:4 dilutions of AL and DR cultures with worms at 0 and 96 hr of culture. There is not a significant change in bacterial density in either AL or DR (p = 0.10, p = 0.19 respectively, paired t-test, n = 3). The data points obscure SEM bars. (TIF) [file pgen.1006396.s001.tif]

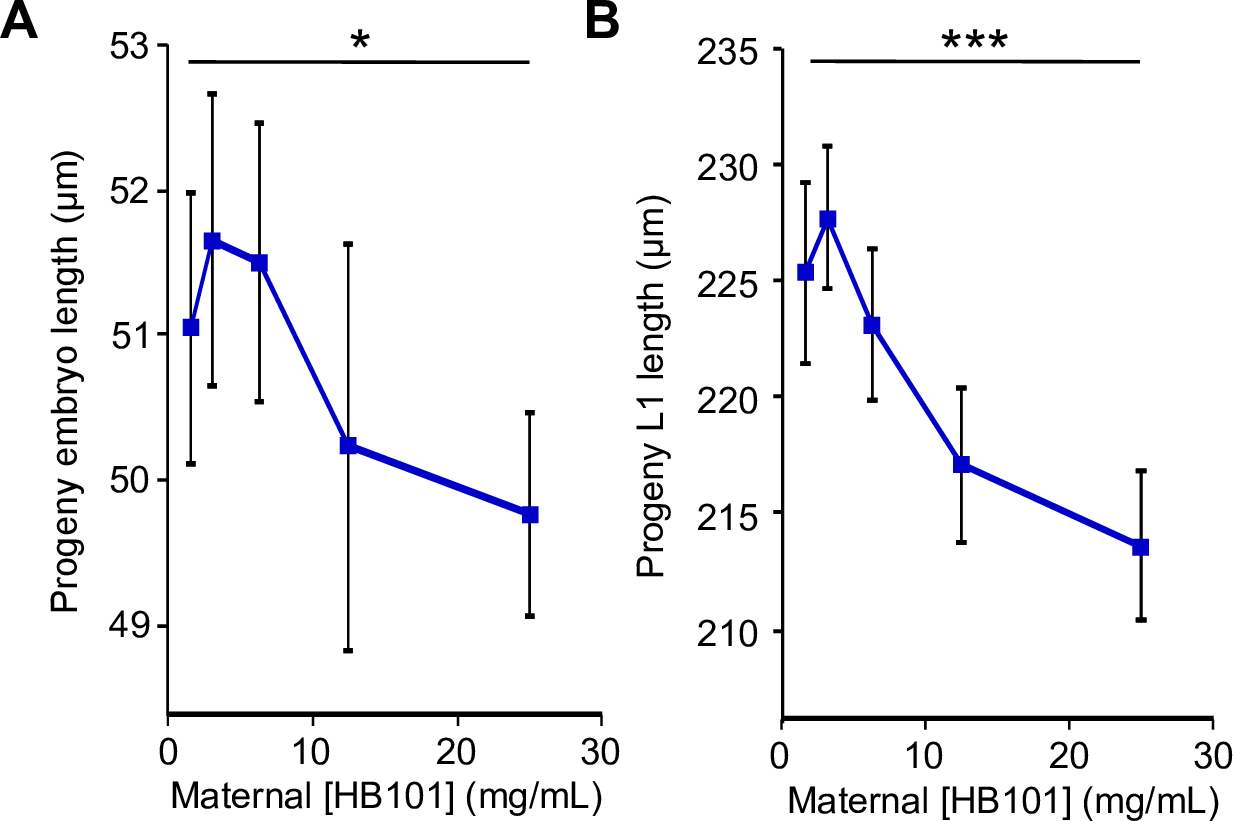

Supplement: S2 Fig — A) Embryo length increases with reduced maternal HB101 (*p = 0.01, 1-way ANOVA, n = 4). B) Progeny L1 length increases with reduced maternal HB101 (***p<0.0001, 1-way ANOVA, n = 6). Mean and SEM are plotted in A and B. (TIF) [file pgen.1006396.s002.tif]

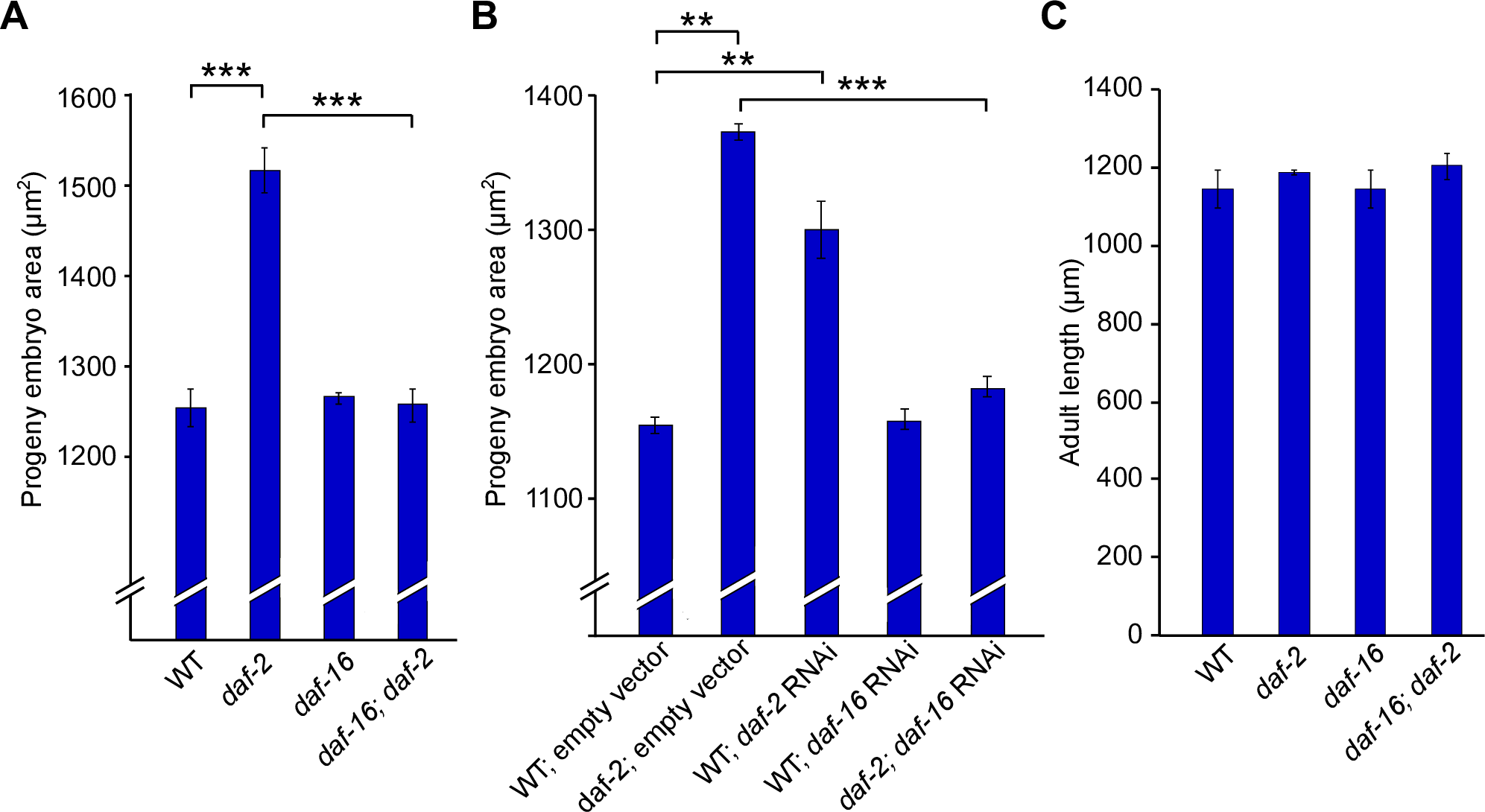

Supplement: S3 Fig — A) Cross-sectional area of embryos is plotted for a variety of genotypes. daf-2(e1370) mutant embryos have significantly greater area than WT (p<0.001, paired t-test, n = 3) and daf-16;daf-2 double mutants (p<0.001, paired t-test, n = 3). daf-16(mgDf47) single mutants and daf-16;daf-2 double mutants are not significantly different from WT. B) Cross-sectional area of embryos is plotted for WT and daf-2 mutants with and without RNAi of daf-2 and daf-16. daf-2 RNAi increases embryo area (p = 0.01, paired t-test, n = 3), consistent with the increased area of daf-2 mutant embryos fed empty vector bacteria (p = 0.003, paired t-test, n = 3). daf-16 RNAi in a daf-2 mutant background suppresses the increase in embryo area (p<0.001, paired t-test, n = 3). C) Adult length is plotted for a variety of genotypes after 96 hr of culture (starting from L1 arrest). Length of WT worms is not significantly different from that of daf-2 (p = 0.49, paired t-test, n = 3), daf-16 (p = 0.24, paired t-test, n = 3), or daf-16;daf-2 double mutants (p = 0.069, paired t-test, n = 3). Mean and SEM are plotted for A-C. (TIF) [file pgen.1006396.s003.tif]

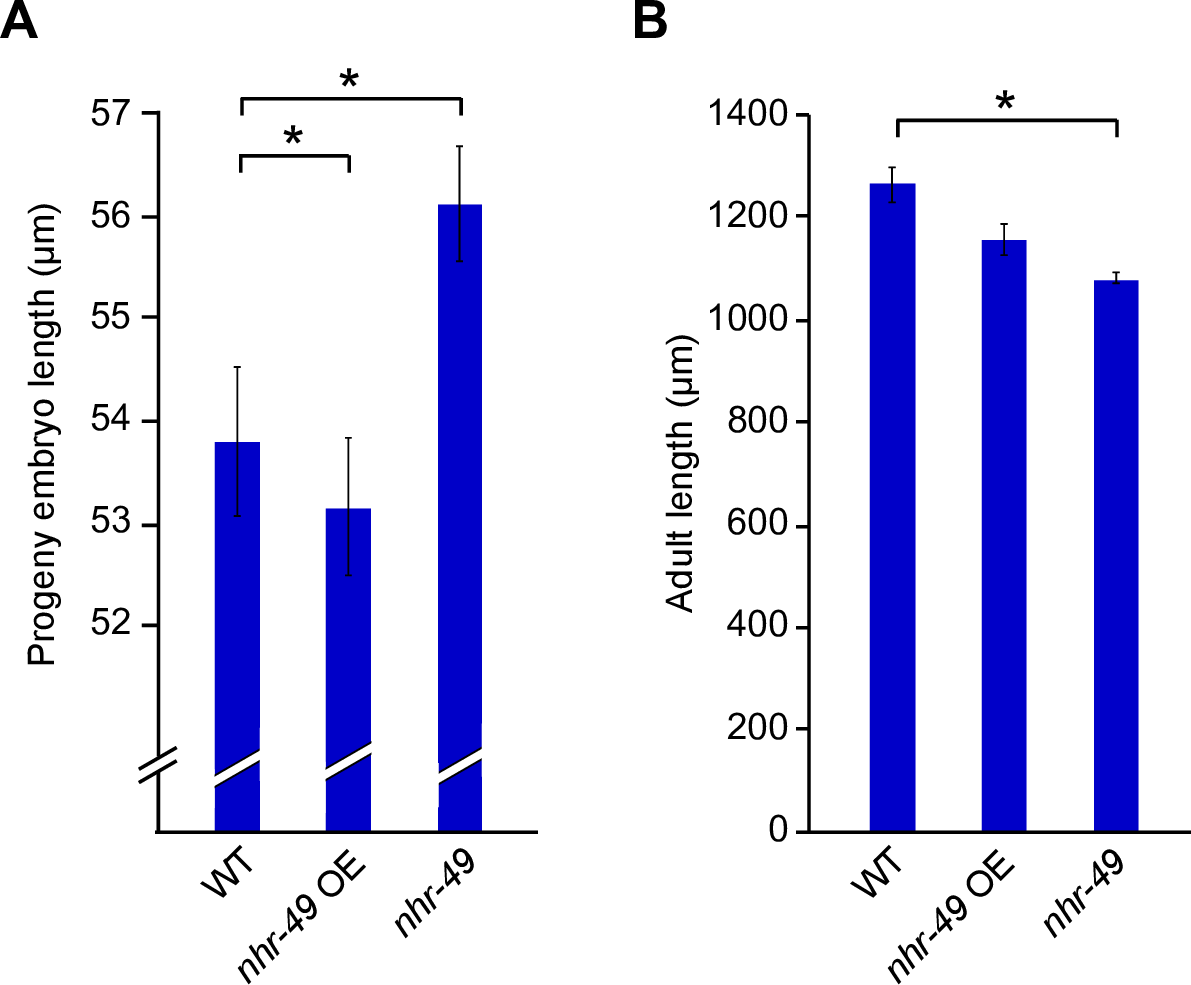

Supplement: S4 Fig — A) Overexpression (OE) of nhr-49 reduces embryo length (p = 0.034, paired t-test, n = 4) and loss-of-function nhr-49 mutation increases embryo length (p = 0.014, paired t-test, n = 4) in AL conditions on plates with OP50. B) Adult length at 96 hr is reduced in an nhr-49 loss-of-function mutant (p = 0.02, paired t-test, n = 3) but not when nhr-49 is overexpressed (p = 0.10, paired t-test, n = 3). Mean and SEM are plotted. (TIF) [file pgen.1006396.s004.tif]

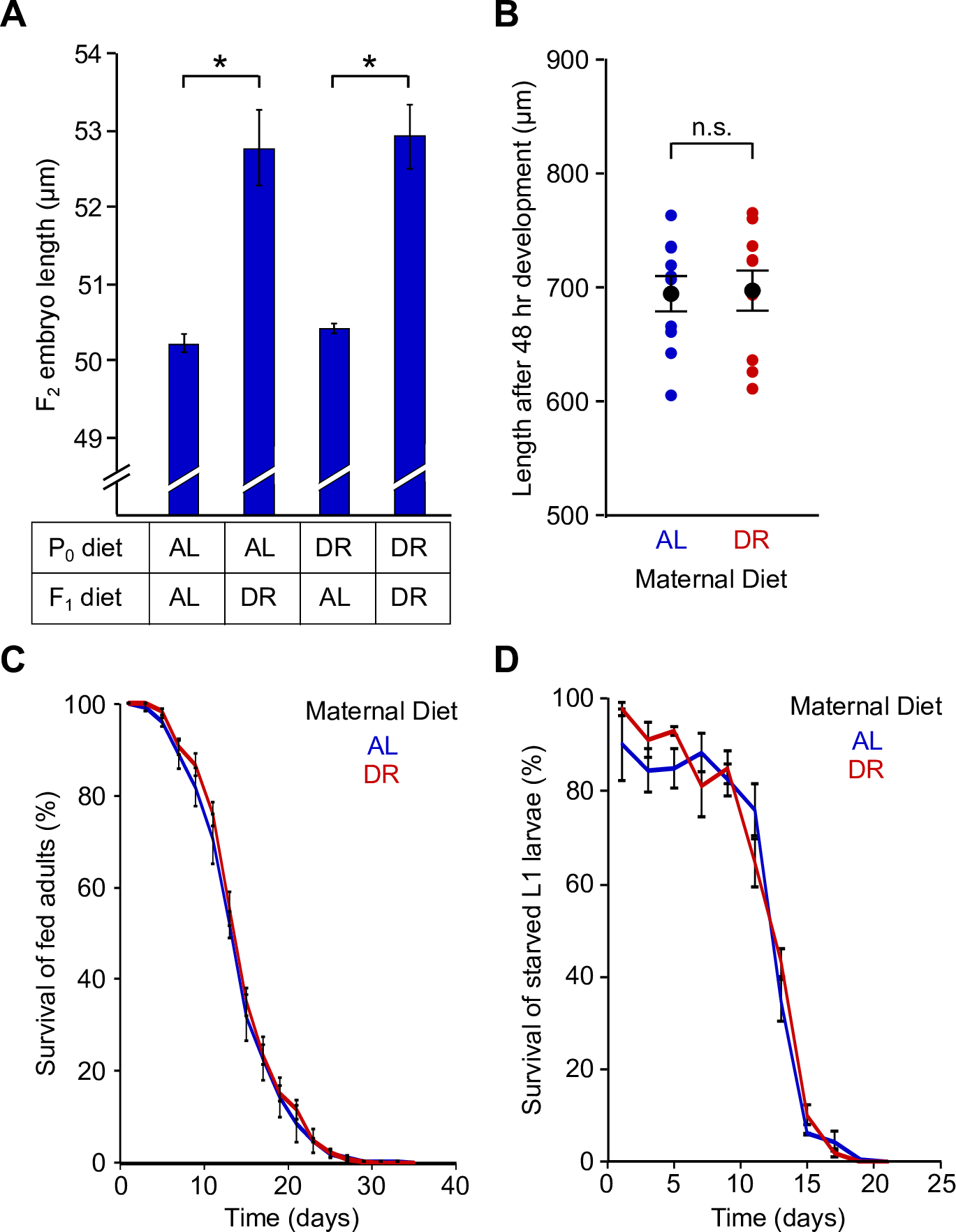

Supplement: S5 Fig — A) Size of embryos produced by progeny of AL or DR mothers is plotted for worms grown in AL and DR conditions. Diet of the F1 progeny, but not their mothers (P0) impacted embryo size (F2 generation) (p = 0.02 in both cases, paired t-test, n = 3) B) Length after 48 hr of postembryonic development (L4-stage larvae) is not significantly different for progeny of AL and DR worms that were not starved (p = 0.72, paired t-test, n = 10). C) Survival of adult worms on plates with OP50 is plotted over time. Progeny of DR worms do not have significantly altered lifespan (p = 0.58, log-rank test, 342 AL animals and 320 DR animals pooled from 3 biological replicates). Although statistics were done on pooled data the mean and SEM of 3 biological replicates are plotted. D) Survival of starved L1 larvae is plotted over time. Progeny of DR worms do not exhibit any difference in L1 starvation survival (p = 0.34, t-test on median survival, n = 4). Mean and SEM of 4 biological replicates are plotted. (TIF) [file pgen.1006396.s005.tif]
