# Supplementary figures and images for: Correction: Maternal Diet and Insulin-Like Signaling Control Intergenerational Plasticity of Progeny Size and Starvation Resistance
Source: PLoS Genet. 2018 Aug 30;14(8):e1007639. doi: 10.1371/journal.pgen.1007639 (PMC6116921; doi:10.1371/journal.pgen.1007639)

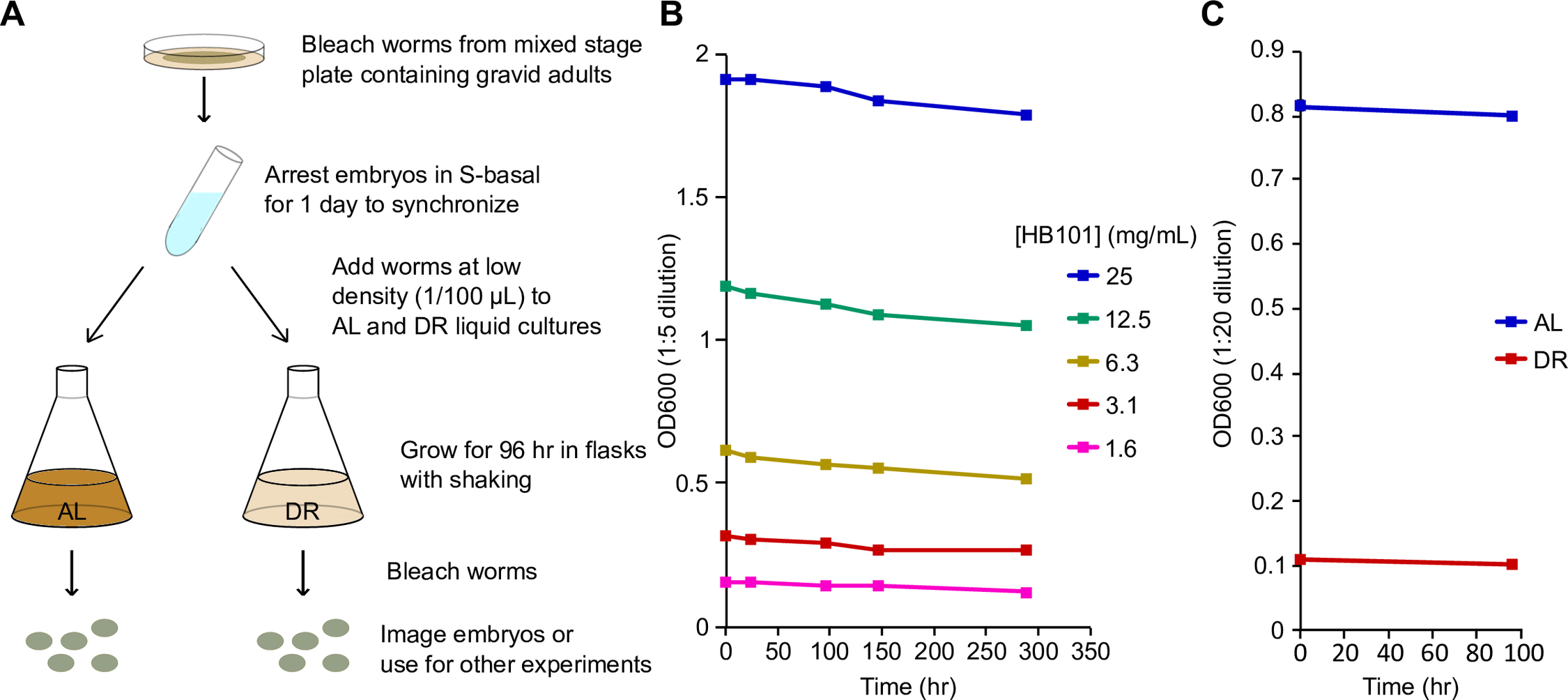

Supplement: S1 Fig — A) Schematic of DR by food dilution in liquid culture. Worms are grown in standard conditions on plates with OP50 and then bleached to obtain embryos. Embryos are hatched in buffer so they enter L1 arrest for synchronization. Arrested L1 larvae are added to culture flasks at a very low density of 10 worms/mL so that they do not reduce bacterial density during culture. E. coli HB101 is used for liquid culture to avoid flocculation. Worms are cultured at 20°C with shaking and typically harvested at 96 hr to collect their embryos for phenotypic analysis. B) Optical density at 600 nm (OD600) is plotted for 1:5 dilutions of different densities of HB101 over time in S-complete, showing that density is roughly constant. C) OD600 is plotted for 1:20 dilutions of AL and DR cultures with worms at 0 and 96 hr of culture. There is not a significant change in bacterial density in either AL or DR (p = 0.10, p = 0.19 respectively, paired t-test, n = 3). The data points obscure SEM bars. (TIF) [file pgen.1007639.s001.tif]
